# Statin as a Potential Chemotherapeutic Agent: Current Updates as a Monotherapy, Combination Therapy, and Treatment for Anti-Cancer Drug Resistance

**DOI:** 10.3390/ph14050470

**Published:** 2021-05-16

**Authors:** Nirmala Tilija Pun, Chul-Ho Jeong

**Affiliations:** College of Pharmacy, Keimyung University, 1095 Dalgubeol-daero, Daegu 42601, Korea; tilijanp@gmail.com

**Keywords:** statin, drug repurposing, anti-cancer, apoptosis, resistance

## Abstract

Cancer is incurable because progressive phenotypic and genotypic changes in cancer cells lead to resistance and recurrence. This indicates the need for the development of new drugs or alternative therapeutic strategies. The impediments associated with new drug discovery have necessitated drug repurposing (i.e., the use of old drugs for new therapeutic indications), which is an economical, safe, and efficacious approach as it is emerged from clinical drug development or may even be marketed with a well-established safety profile and optimal dosing. Statins are inhibitors of HMG-CoA reductase in cholesterol biosynthesis and are used in the treatment of hypercholesterolemia, atherosclerosis, and obesity. As cholesterol is linked to the initiation and progression of cancer, statins have been extensively used in cancer therapy with a concept of drug repurposing. Many studies including in vitro and in vivo have shown that statin has been used as monotherapy to inhibit cancer cell proliferation and induce apoptosis. Moreover, it has been used as a combination therapy to mediate synergistic action to overcome anti-cancer drug resistance as well. In this review, the recent explorations are done in vitro, in vivo, and clinical trials to address the action of statin either single or in combination with anti-cancer drugs to improve the chemotherapy of the cancers were discussed. Here, we discussed the emergence of statin as a lipid-lowering drug; its use to inhibit cancer cell proliferation and induction of apoptosis as a monotherapy; and its use in combination with anti-cancer drugs for its synergistic action to overcome anti-cancer drug resistance. Furthermore, we discuss the clinical trials of statins and the current possibilities and limitations of preclinical and clinical investigations.

## 1. Background

Cancer, a chronic disease, is a leading cause of death worldwide [1,2] and seriously threatens human health [3,4]. Different technologies such as surgery, radiotherapy, chemotherapy, targeted therapy, radiopharmaceutical therapy, gene therapy, and cancer immunotherapy have been developed as modes of treatment, and most of them reach clinical trials [5,6,7,8]. Despite the tremendous research and resources being investigated to prevent and cure cancers, they remain incurable owing to metastasis, recurrence, and resistance [9]. Therefore, it remains necessary to develop additional technologies or drugs to reduce the rate of cancer-caused deaths. However, the successful translation of new drugs and technologies from the development phase to clinical practice requires an average of 13 years of research with an investment of USD $1.8 billion [10,11]. This is because preclinical and clinical studies are a key step in testing the safety and efficacy of a new drug in humans [12] for use. After the discovery of a new drug, it undergoes preclinical testing, including evaluation in vitro (cell culture) and in vivo (animal) models for determining the preliminary safety, efficacy, and pharmacokinetics of a drug. When its preclinical study is approved, it further undergoes clinical trials in human subjects; these trials comprise phase I (a small group of people, 20–100), II (a larger number of people, 20–300), III (a large group of patients, 300–3000), and IV (the largest group of patients, ˃3000). Altogether, clinical trials include testing a drug for its safety, tolerability, pharmacokinetics, and pharmacodynamics. Following a phase III clinical trial, a drug can be approved in the United States by the Food and Drug Administration (FDA) or in the European Union by the European Medicines Agency (EMEA). Phase IV studies occur after the approval of a drug and are marketed for use over a longer period while still being monitored [10,12,13]. However, most drugs fail in their phase II clinical trial [14], and only 8% of new molecular entities (NMEs) successfully progress from the pre-clinical stage to the final launch [10].

Consequently, the concept of drug repurposing (in other words “new indications of old drug”) evolved to enable shortening of the development cycle and saving resources in drug discovery and development, along with a reduction in the risk of failure in early clinical trials. This concept involves utilization of an existing clinically approved drug for a novel indication so that a new mode of action can be applied for that condition [15]. On the basis of this concept, several researchers and clinicians are focusing on drugs approved for use in cancer to fulfill the need for new cancer therapies [16,17,18]. The advantage of this approach is that the documentation of the pharmaceutical agent is available with a history of clinical use (pharmacokinetics, bioavailability, toxicity, protocol, and dosing) established in phase I clinical studies. Therefore, these drugs could be rapidly advanced into phase II and phase III, and the associated cost and time could be significantly reduced [19].

Drug repurposing approaches are achieved either experimentally or through a computational method by identification of drugs with new indications, new targets, new effects, and unexplored mechanisms [20]. It has received increasing attention not only from the pharmaceutical industry but also from the public sector and academia, as it is a faster and cheaper strategy for enlarging the collection of approved drugs. Furthermore, it has gained acceptance with respect to cancer treatment as cancer is difficult to cure, and it takes a long time for a new drug to be discovered and implemented. Different drugs available in the market have been repurposed in cancer therapy, such as cardiovascular drugs, antipsychotic and antidepressant drugs, microbiological agents, antiviral drugs, antibiotics, nonsteroidal anti-inflammatory drugs (NSAIDs), aspirin, metformin, selective estrogen receptor modulators (SERMS), and statins [21].

Cancer resistance, which is characterized by tumor relapse or spread, remains a major challenge in clinical oncology; its occurrence is attributed to unsuccessful adaptation and evaluation of the treatment modality [3,5]. Chemotherapeutic resistance is mediated by two mechanisms: intrinsic and acquired resistance. Intrinsic resistance occurs before treatment because of resistance-mediating factors that exist in the bulk of tumors, which later renders cancer therapy inefficient. In contrast, acquired resistance develops during or after treatment of tumors that are sensitive initially but can develop mutations later or after adaptive responses such as mutation, drug efflux, drug activation/inactivation, and alteration of the drug target [4,22]. Several drugs such as immunomodulatory drugs, antihypertensives (calcium channel blockers, angiotensin II receptor inhibitors, diuretics, and β-blockers), antidiabetics (biguanides and thiazolidinediones), anthelmintics (niclosamide, mebendazole, albendazole, and ivermectin), antimalarials (chloroquine and hydroxychloroquine; primaquine; and mefloquine, amodiaquine, and artemisinin derivatives), anti-fungals (itraconazole), antibiotics (anisomycin, monesin, salinomycin, gramicidin A, and minocycline), and antivirals (brivudine and phenothiazines) have been considered potentially to be repurposed to resensitize multi-drug resistant cancers to conventional chemotherapeutic agents [23]. Therefore, the concept of drug repurposing is a potential strategy in overcoming chemotherapeutic resistance.

In this review, we discuss the possibility of repurposing statins for various cancers. We particularly focus on different possible approaches for using statins, such as an anti-cancer monotherapy in combination with other chemotherapeutic drugs for achieving a synergistic effect, as well as in combination with other chemotherapeutic drugs for overcoming resistance. Additionally, we discuss the effect of statins on resistance development and tolerance, highlight relevant signaling pathways associated with statins, and briefly describe clinical studies on repurposing statins.

## 2. The Emergence of Statin as a Lipid-Lowering Drug

Cholesterol is essential for the functioning of all organs in humans; however, an elevated cholesterol level is a risk factor for atherosclerosis and coronary heart disease. 3-Hydroxy-3-methyl-glutaryl-CoA (HMG-CoA) reductase is the rate-limiting enzyme in the cholesterol biosynthesis pathway [24]. Therefore, the inhibition of HMG-CoA reductase is needed for reducing cholesterol synthesis in the treatment of atherosclerosis and coronary heart disease, which were the leading causes of death in 1950. In 1950 and 1960, several companies and researchers explored molecules that could block a step of cholesterol synthesis, which is comprised of 30 steps; consequently, compactin/mevastatin was discovered in 1967 as the first statin that strongly inhibited HMG-CoA reductase to lower serum cholesterol levels [25,26]. However, owing to the development of serious side effects such as flushing, gastrointestinal (GI) discomfort, gallstone, liver disease, and cataract, it was removed from the market [26]. In 1979, two research groups, Merck Research Laboratory and Tokyo University of Agriculture and Technology, isolated statins named mevinolin and monocolin K from *Aspergillus terreus* and *Monasus ruber*, respectively. However, as mevinolin and monocolin K were determined to be the same compound, it was named lovastatin, which later became the first commercial statin in September 1987 [24,25,26].

**Figure 1:**
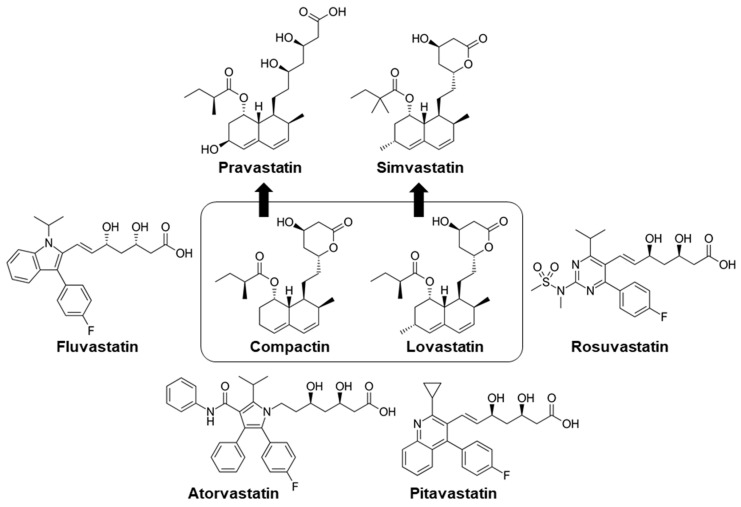
Chemical structure of all major statins (derived from [25]).

Due to the issue of optimal clinical activity of lovastatin and the lack of patent protection in many countries, the efficacy of lovastatin led to the discovery of simvastatin, which is more potent than lovastatin [27]. As is the case for all discovered statins, compactin, lovastatin, and simvastatin are closed ring structures, meaning they are inactive prodrugs that need reopening for the activity. This has led to the discovery of open ring structure statins, i.e., pravastatin, fluvastatin, atorvastatin, rosuvastatin, and pitavastatin (Figure 1) [27,28]. In 1989, atorvastatin was discovered as the first synthetic statin with a potent novel hypolipidemic effect; it was marketed in the United States as LIPITOR [29]. Later, it was recommended as the first-line therapy for patients with low to high risk of coronary heart disease, as it reduces the risk of cardiovascular morbidity and mortality [30].

The use of statin is not beyond side effects. After the use of statin, the patient suffers from liver toxicity although the incidence of this is low. Moreover, statin has increased the incidence of diabetes risk by 20–30%. Myopathy is also another frequent toxicity encountered in daily practice, although the nature of muscle pain is unclear. Other potential toxicities such as proteinuria and hematuria have been described as well (Table 1) [27].

## 3. Statin Repurposed in Cancer Therapy

Being overweight or obese is linked to the development and recurrence of breast cancer [31]. Tumor cells show high avidity for cholesterol, as well as an accumulation of intracellular cholesterol supports proliferation, growth, and metastasis of cancers [32,33]. Furthermore, the inhibition of the mevalonate pathway, a metabolic pathway of cholesterol synthesis, or lowering of cholesterol, has been proven to prevent cancer progression [34,35]. Statin, a drug that inhibits the rate-limiting step of the mevalonate pathway, particularly the HMG-CoA reductase enzyme, has gained interest for its use in overcoming mycobacterial infection, insulin, and cancer resistance [36,37,38]. In addition, the inhibition of the cholesterol biosynthetic pathway has shown a promising effect in reducing the formation of mammospheres enriched with cancer stem cells, indicating that the cholesterol biosynthetic pathway is a potential therapeutic target for statin treatment in breast cancers [39].

Other statins such as simvastatin, atorvastatin, and rosuvastatin suppress geranylgeranylation and expression of transforming growth factors (TGF-β1); vascular endothelial growth factor (VEGF); and the tumor-promoting cytokines and mediators IL-6, IL-8, and TNF-α, inhibiting ovarian cancer cell growth [40]. Statins have been used as a single agent to decrease cell proliferation and to induce apoptosis in many cancer cells. Moreover, they have been used in combination with other chemotherapeutic drugs for improving the efficacy of drugs and the condition of the patient. For example, clinical data shows that the concomitant use of statins and trastuzumab lowers the cardiotoxicity of trastuzumab-based therapy in HER2-positive breast cancers [41]. Inhibition of the mevalonate pathway using statins (pitavastatin, simvastatin, lovastatin, atorvastatin, pravastatin, and rosuvastatin) inhibits radiation resistance in head and neck cancers, indicating that the mevalonate pathway can serve as a vital target for overcoming resistance development [42]. Moreover, statins in combination with metformin have been shown to decrease the all-cause mortality of prostate cancer patients at high risk, particularly in post-diagnostic settings [43].

**Table 1:** Clinical trials showing the use of statins with their clinical indications, toxicities, doses, and human plasma concentration.

S.N.	Statin	Clinical Indication	Doses	Human Plasma Concentration	Toxicity	Ref
1	Lovastatin	Multiple myeloma	2 mg/kg days 1–5, 8–12 and 0.5 mg/kg days 15–28 of each cycle	-	Somnolence, fatigue and constipation, deep vein thrombosis, pulmonary embolism	[44,45]
2	Simvastatin	Refractory multiple myeloma, pancreatic cancer, colorectal cancer,	30 mg, 80 mg daily	-	Hematoxicity, bone pain, gastrointestinal side effects, infections, muscle pain, fatigue, anemia, depression	[46,47,48]
3	Pravastatin	Gastric cancer, hepatocellular carcinoma (HCC)	20–40 mg/kg	-	Diarrhea, stomatitis	[49]
4	Fluvastaatin	Prostate cancer	80 mg	63.4 ng/mL or 0.2 μM (0.0–437.0 ng/mL or 0.0–1.1 μM)	-	[50]
5	Atorvastatin	Prostate cancer	80 mg	3.6 ng/mL	-	[51]
6	Rosuvastatin	Advanced solid malignancies	20 mg, 80 mg daily	-	Fatigue, myalgia, muscle weakness	[52,53]

## 4. Statin as a Single Agent in the Suppression of Cancer Cell Proliferation, and the Induction of Apoptosis

**Figure 2:**
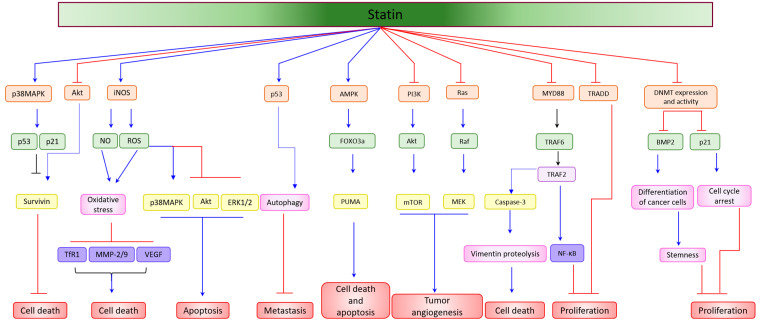
Statins alone as anti-cancer agents. Statin as a monotherapy upregulates or inhibits diverse signaling cascades leading to induction of oxidative stress, cell-cycle arrest, differentiation of cancer cells, autophagy, and suppression of cancer stemness, proliferation, metastasis, angiogenesis. As a result, statin induces cell death, cytotoxicity, and apoptosis of cancer cells. Blue arrows indicate upregulation, red colored lines indicate inhibition/suppression.

Statin was studied in vitro for its anti-proliferative and apoptotic effect in cancer cells such as medulloblastoma brain tumor, colorectal cancer, lung cancer, oral squamous cell carcinoma, anaplastic thyroid cancer, and hepatic cancer (Figure 2) [54,55,56,57,58,59,60]. Lovastatin augments sensitivity by activating bone morphogenetic protein (BMP), a tumor-suppressive protein, and reducing cancer stemness in colorectal cancer cells [61]. The anti-cancer effect of statin is also mediated by inhibiting the activity of DNA methyltransferases (DNMTs) [60,61], leading to demethylation and activation of BMP signaling, which causes a shift in the stem-like state to a differentiated form of cancer cells [61] and induction of p21 ^cip^, causing cell-cycle arrest [60]. In contrast, it inhibits breast cancer cell proliferation via induction of cell-cycle arrest and apoptosis [62,63]. Simvastatin has been shown to modulate intrinsic and extrinsic apoptosis simultaneously in prostate cancer cells. It enhances phosphorylated Bad, and cleavage of caspases 9/3 but reduces Bcl-2 and Bcl-xL as intrinsic apoptotic markers. While as extrinsic apoptosis, it increases TNF, Fas-L, Traf, and caspase 8 cleavage [64]. It also induces cholangiocarcinoma cancer cell death by disrupting the colocalization of Rac1/lipid rafts, depressing Rac1 activity, and suppressing the expression of ATP-binding cassettes (ABCA1 and ABCG1) [65,66]. The anti-cancer effect of simvastatin in salivary adenoid cystic carcinoma (SACC) is further enhanced by the inhibition of microRNA-21, as it is highly expressed in tumors and promotes tumor development [67]. A clinical study conducted in Taiwan National Health Insurance Research Database (NHIRD) in a cohort of 15,264 hyperlipidemic prostate cancer patients shows that the mortality rate was decreased in patients receiving simvastatin or lovastatin [68]. Additionally, simvastatin improves the radiosensitivity of esophageal cancers by inducing the tumor suppressor protein PTEN and inhibiting the tumor-promoting signaling PI3K/Akt pathway, leading to a decrease in proliferation, invasion, migration, and induction of apoptosis [69].

Pravastatin suppresses the viability of multiple myeloma cells by decreasing the production of growth factors such as VEGF and bFGF, and inducing cell-cycle arrest [70]. Furthermore, it hinders the proliferation and invasion of human HCC cells [71]. It is an effective anti-fibrotic agent that reverses radiation-induced fibrosis of head and neck cancers [72]. Fluvastatin hinders the glycosylation of FLT3 in human and murine cells, acting as an anti-cancer agent and prolonging the survival of FLT3/ITD leukemic mouse models [73]. Fluvastatin inhibits cancer cell proliferation and induces apoptosis in cancer cells such as breast cancer, cervical cancer, glioma, and lymphoma [74,75,76,77,78,79]. Fluvastatin, cerivastatin, and pitavastatin have demonstrated potent anti-proliferative effects along with the induction of autophagy in primary glioblastoma cell lines [80]. In a nude mouse model, fluvastatin prevented lung adenocarcinoma bone metastasis, which is largely dependent on p53-mediated autophagy induction [81]. Fluvastatin conjugated with human immunodeficiency virus type 1 (HIV-1) trans-activator transcription peptide (TAT) produces anti-proliferative action against human hepatoma cancer cells through a concomitant accumulation of cells in the pre-G phase and induction of caspase 3 cleavage [82]. Atorvastatin causes strong growth inhibition of epithelial- and mixed epithelial-mesenchymal cancer cells by inhibiting the protein prenylation pathway [83]. Rosuvastatin inhibits cell proliferation and spheroid formation without cytotoxicity in prostate cancer cells and inhibits the expression of EMT markers vimentin, and Zeb-1 [84]. Novel small molecules based on isocoumarins/3-alkylidenephthalides that were derived from rosuvastatin have shown promising properties for use as anti-cancer drugs in the future [85]. Furthermore, gel-loaded rosuvastatin [86], fabrication of rosuvastatin [87], and biocompatible copolymeric micelles of rosuvastatin [88] have shown an anti-cancer effect in aggressive tongue carcinoma, hepatic cancer, and breast cancer, respectively, with improved efficacy compared to rosuvastatin alone. Pitavastatin treatment induces cell death in ovarian cancer cells in which hydroxymethylglutarate coenzyme-A reductase (HMGCR) is upregulated and TP53 is mutated, and the anti-cancer effect of pitavastatin is solely mediated by the inhibition of HMGCR [89]. Pitavastatin further inhibits AKT activation while activating AMPK, which leads to FOXO3A activation and induction of PUMA, ultimately inducing apoptosis in oral squamous cancer [90]. Pitavastatin overcomes radiotherapy resistance in breast cancer and melanoma models by increasing DNA double-strand breaks [91]. Cerivastatin inhibits proliferation, migration, invasion, metastasis, and angiogenesis in several cancers such as aggressive breast cancers and glioblastoma [92,93,94,95,96].

## 5. Statins Possess Synergistic Action to Overcome the Resistance to Anti-cancer Therapies

**Figure 3:**
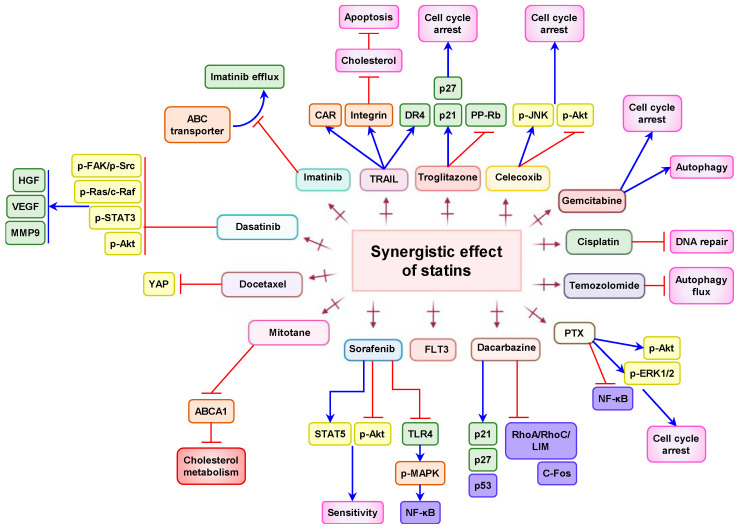
Synergistic action of statins. Statin in combination with anti-cancer drugs such as imatinib, TRAIL, troglitazone, celecoxib, gemcitabine, cisplatin, temozolomide, PTX, dacarbazine, FLT3, sorafenib, mitotane, docetaxel, and dasatinib synergistically suppress and induce signaling cascade leading to cell-cycle arrest, cell death, apoptosis, and sensitivity. Blue arrows indicate upregulation, black colored lines indicate inhibition/suppression, and red arrows indicate the combination of statin with indicated drugs.

**Figure 4:**
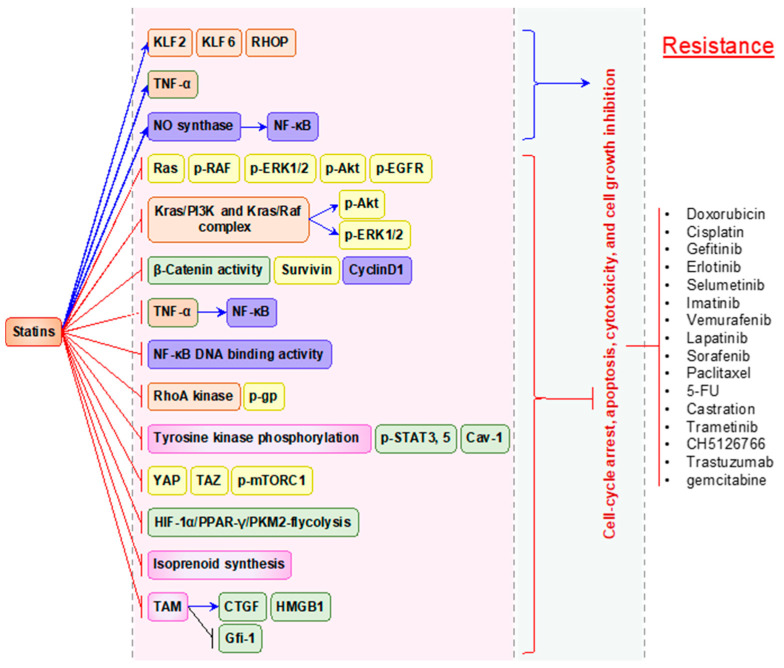
Statins for overcoming anti-cancer drug resistance. Statin overcomes the resistance developed by various anti-cancer drugs (as indicated in the figure) through the induction of cell-cycle arrest, apoptosis, cytotoxicity, and inhibition of cell growth. In most cases, statin inhibits the signaling molecules or kinases involved in cancer cell proliferation, growth, metastasis, angiogenesis, inflammation, and multi-drug resistance mechanism developed by those anti-cancer drugs. Blue arrows indicate upregulation, red colored lines indicate inhibition/suppression.

We next discuss the in vitro synergistic effects of statins with other anti-cancer drugs (Figure 3) and their potential for overcoming drug resistance to different chemotherapeutic drugs (Figure 4). These are summarized in Table 2.

### 5.1. Doxorubicin

Doxorubicin (DOX), isolated from *Streptomyces* spp., is a topoisomerase II inhibitor belonging to the family of anthracycline anticancer drugs that breaks the DNA chain for replication, stopping the process of replication [97]. It is used for the treatment of wide range of cancers. However, the resistance among cancer cells has emerged as a major barrier to effective treatment using DOX. Simvastatin induces glutathione (GSH)-mediated suppression of ABCG4 (which causes efflux of intracellular doxorubicin and cisplatin) levels, increasing the sensitivity of prostate cancer cells to doxorubicin or cisplatin and leading to the suppression of tumor growth and size without doxorubicin-induced cytotoxicity [98]. An in vivo tumor xenograft model of breast cancer showed that encapsulation of fluvastatin in a hyaluronan-conjugated liposome and administration with doxorubicin produced a potent anti-proliferative effect with the longest survival in mice [99]. Atorvastatin sensitizes the anti-cancer effect of doxorubicin and cisplatin in human osteosarcoma by suppressing matrix metalloprotease 2 (MMP2) induced by doxorubicin and cisplatin [100]. Cerivastatin enhances the anti-tumor activity of doxorubicin and cisplatin against human breast cancer cells [101].

Among statins, simvastatin has been well studied in the context of overcoming chemotherapeutic resistance. An initial study was performed in doxorubicin, in which simvastatin and mevastatin treatment effectively improved doxorubicin resistance in human malignant mesothelioma (HMM). As the development of doxorubicin resistance is caused by the efflux of it by drug efflux mechanism, decrease in doxorubicin resistance is mediated by the inactivation of the ABC transporter P-glycoprotein (P-gp) by nitric oxide (NO)-dependent nitration of a tyrosine residue of P-gp [102]. Further, the combination of simvastatin with phenothiazine derivatives improves resistance to doxorubicin in colon cancer cells by suppressing the mRNA and protein expression of multi-drug resistance protein, P-gp, and inflammation markers Cox-2 [103]. Similarly, simvastatin has been observed to decrease multidrug resistance (MDR1) protein and P-gp in leukemic cells, and a decrease in its expression is an additional mechanism underlying the simvastatin-mediated increase in chemosensitivity and overcoming of drug resistance [104]. Doxorubicin mediates resistance in chronic lymphocytic leukemia (CLL) by upregulating RhoA/RhoA kinase, Ras/ERK1-2, Akt, HIF-1α, and P-gp activities; simvastatin inhibits this effect, overcoming doxorubicin resistance [105]. Doxorubicin has been observed to develop resistance in urothelial bladder cancer by inducing the mevalonate (cholesterol) biosynthetic pathway; simvastatin inhibits the mevalonate pathway, thus reversing doxorubicin resistance [106]. Furthermore, simvastatin overcomes doxorubicin-mediated resistance of colon cancer cells by potentiating the anti-cancer activity of oxicam derivatives, leading to apoptosis induction and suppression of cell survival [107]. In addition, simvastatin and mevastatin in combination with flavonoids inhibited doxorubicin resistance in colon cancer cells [108]. Different statins have shown different behaviors in terms of overcoming drug resistance. Researchers evaluated the effect of natural statins (lovastatin, simvastatin, mevastatin, and pravastatin) and synthetic statins (atorvastatin and fluvastatin) on the chemoresistance of chemotherapeutic drugs such as doxorubicin, paclitaxel, and 5-fluorouracil (5-FU) in human myeloid leukemia cells. For example, natural statins such as simvastatin, mevastatin, lovastatin, and pravastatin have shown promising effects on enhancing the cytotoxicity of doxorubicin, paclitaxel, and 5-fluorouracil (5-FU) compared with synthetic statins (fluvastatin and atorvastatin) through the suppression of NF-κB activity in human myeloid leukemia [109]. YAP and TAZ oncoproteins induce drug resistance (doxorubicin and paclitaxel) in various cancers. Fluvastatin has been shown to improve resistance to doxorubicin and paclitaxel in breast cancer cells, which exhibit a higher expression of YAP and TAZ. Overcoming the resistance is attributed to the inhibition of target genes and nuclear localization of YAP and TAZ [110]. Furthermore, the combination of statins (fluvastatin and atorvastatin) with chemotherapeutic drugs such as doxorubicin, paclitaxel, or topotecan inhibits cell proliferation and increases toxicity in leukemia cells; this effect is due to the inhibition of ERK MAP kinase [111].

### 5.2. 5-Fluorouracil (5-FU) and Capecitabine

5-fluorouracil is an antimetabolite that inhibits the cell growth by interfering with DNA synthesis and mRNA translation [112]. Lovastatin increases the efficacy of 5-fluorouracil (5-FU) even at low doses (1–10 µM); 5-FU at this dose is unable to inhibit the viability of colorectal cancer cells [61], and the dose of 5-FU at 1–10 µM is clinically relevant in a patient, resulting in an approximate level of 6–12 µM in the serum [113] and 2–5 µM in tissues [114]. Cerivastatin is cytotoxic to colorectal cancer cells that are both sensitive and resistant to 5-fluorouracil (5-FU); however, higher efficacy is observed in resistant cells, and the efficacy of cerivastatin in overcoming 5-FU-resistance is partially independent of the mevalonate pathway [115]. In 5-FU-resistant colorectal cancer cells, simvastatin synergistically sensitized colorectal cancer cells to fluorouracil treatment by inducing diverse actions such as anti-inflammation, anti-angiogenic, antioxidant, and by inhibiting tumor metastasis and invasion [116].

Simvastatin sensitizes gastric cancer to capecitabine in human gastric cancer xenografts by inhibiting NF-κB activation and abrogation of cyclin D1, cyclooxygenase-2 (COX-2), survivin, Bcl-2, CXC motif receptor 4, and MMP-9 [117].

### 5.3. Sorafenib

Sorafenib is a protein kinase inhibitor with activity against diverse protein kinases such as VEGFR, PDGFR, and RAF [118]. Fluvastatin enhances the cytotoxic effect of sorafenib by modulating JNK and Akt signaling in melanoma cells [119]. In hepatocellular carcinoma (HCC), hypoxia causes sorafenib resistance by activating YAP, leading to the upregulation of survival genes [120]. Fluvastatin combination with sorafenib inhibits hepatic stellate cell activation and Toll-like receptor 4 (TLR-4)-mediated MAPK and NF-κB activation and decreases stromal cell-derived factor 1α, leading to a decrease in cell viability and induction in apoptosis [121]. Atorvastatin also ameliorated hypoxia resistance and increased the sensitivity of hypoxic HCC cells to sorafenib treatment [120]. Further, the treatment with simvastatin has improved sorafenib resistance via suppression of HIF-α1/PPAR-γ/PKM2 signaling [122]. In a phase II clinical trial, a combination of pravastatin with sorafenib was safe and well-tolerated with prolonged time to progression (TTP) in advanced hepatocellular carcinoma [123].

### 5.4. Gefitinib, Erlotinib, and Imatinib

Gefitinib acts by inhibiting EGFR and the amplification and mutation of EGFR in glioblastoma render it resistant to the clinical effects of the EGFR inhibitor gefitinib and the EGFR-targeting antibody cetuximab [124,125]. Targeting HMG-CoA using lovastatin enhances the sensitivity of glioblastoma cells to gefitinib, and the synergistic effect was found to be independent of the status of EGFRvIII and PTEN [126]. In vitro and in vivo studies of human cholangiocarcinoma showed that lovastatin overcomes gefitinib resistance by upregulating tumor necrosis factor-α (TNF-α) [127]. Mutation in the K-Ras gene accounts for 20% to 30% of non-small cell lung carcinomas (NSCLCs) associated with gefitinib resistance [128], and administration of lovastatin with gefitinib inhibits growth and induces apoptosis and DNA fragmentation by downregulating RAF/ERK and the Akt pathway [129].

EGFR mutation is also the major cause of drug resistance in non-small cell lung carcinoma treated with inhibitors; gefitinib and erlotinib because of the induction of survivin and survivin-mediated mutation at T790 of EGF. Simvastatin overcomes this resistance and induces apoptosis by suppressing Akt/β-signaling [130]. Furthermore, it restores gefitinib-suppressed BIM expression, increasing sensitivity to gefitinib in non-small cell lung carcinoma [131]. Atorvastatin reverses gefitinib resistance in KRAS-mutant non-small cell lung carcinoma, irrespective of PIK3CA and PTEN status [132].

Both statins (pitavastatin and fluvastatin) activate apoptosis in non-small cell lung carcinoma (NSCLC), and the combination of pitavastatin with EGFR tyrosine kinase inhibitor (erlotinib) synergistically increased pitavastatin cytotoxicity in K-ras mutated cells [133].

Lovastatin increases the sensitivity and efficacy of imatinib in melanoma by blocking the ABC transporter-mediated efflux of imatinib, which leads to an increase in the intracellular level of imatinib [134]. Simvastatin shows higher efficacy in imatinib-resistant chronic myelogenous leukemia cells than in sensitive cells via suppression of tyrosine phosphorylation and activation of STAT3 and STAT5, leading to cell-cycle arrest and apoptosis induction [135].

### 5.5. Cisplatin

Cisplatin interlinks with the purine bases on the DNA; interfering with DNA repair mechanisms, causing DNA damage [136]. Simvastatin induces glutathione (GSH)-mediated suppression of ABCG4 (which causes efflux of intracellular doxorubicin and cisplatin) levels, increasing the sensitivity of prostate cancer cells to cisplatin and leading to the suppression of tumor growth [98]. Atorvastatin sensitizes the anti-cancer effect of cisplatin in human osteosarcoma by suppressing matrix metalloprotease 2 (MMP2) induced by cisplatin [100]. Cerivastatin enhances the anti-tumor activity of cisplatin against human breast cancer cells [101]. Along with cisplatin, lovastatin synergistically suppressed gall bladder cancer growth through inhibition of the mevalonate pathway [137]. In cisplatin-resistant prostate and cervical cancer cells, lovastatin overcomes resistance by upregulating tumor suppressor genes such as Ras homolog family member B (RHOB) and kruppel-like factor 2 (KLF2) and 6 (KLF6) [138]. Pitavastatin significantly enhances the efficacy of cisplatin in lung cancer cells and its tumor xenograft model without causing toxicity in mice; this effect is mediated by the suppression of Ras/Raf/MEK and PI3K/Akt/mTOR signaling [139].

### 5.6. Gemcitabine

Gemcitabine inhibits the DNA synthesis process [140]. Pancreatic cancer cells show poor response to gemcitabine treatment. However, treatment with gemcitabine in combination with statins, such as simvastatin, atorvastatin, rosuvastatin, fluvastatin, pitavastatin, and pravastatin is an effective treatment for pancreatic cancers, particularly gemcitabine-resistant cancer [141]. Furthermore, it synergistically improved the anti-cancer efficacy of gemcitabine in human cholangiocarcinoma cells [142]. The synergistic anti-cancer effect of gemcitabine and pitavastatin on pancreatic ductal adenocarcinoma (PDAC) is mediated by cell-cycle arrest at sub-G1 and S phases, leading to downregulation of cyclin A2/CDK2 and upregulation of p21/p27. Furthermore, activated autophagy was observed to be involved in the cell death mechanism [143]. Pancreatic ductal adenocarcinoma (PDAC) resistance to gemcitabine is attenuated by simvastatin via suppression of TAM-mediated suppression of Gfi-1 and induction of CTGF and HMGβ1 [144].

### 5.7. Vemurafenib

Vemurafenib is the selective inhibitor of BRAF kinase leading to the aberrant mitogen-activated protein kinase (MAPK) pathway [145]. Simvastatin has shown promising effects in overcoming drug resistance to diverse anticancer drugs in different cancer cells. For example, simvastatin, by inhibiting RAS or BRAF signaling, reversed vemurafenib resistance developed through activation of RAS or BRAF in mutant melanoma [146]. Simvastatin improved the inefficiency of vemurafenib (BRAF inhibitor) or selumetinib (MEK inhibitor) as an anti-cancer agent in MAPK mutant melanoma by inhibiting isoprenoid synthesis [147]. The efficacy of vemurafenib in the treatment of metastatic melanoma has been decreasing owing to the development of resistance, which was observed to be inhibited by fluvastatin treatment. The inhibitory effect of fluvastatin on vemurafenib resistance is mediated by the inhibition of the PI3K/Akt pathway [148].

### 5.8. TRAIL

Lovastatin synergistically enhances the efficacy of TNF-related apoptosis-inducing ligand (TRAIL) in human refractory prostate cancer cells via the upregulation of death receptor 4 (DR4) [149]. In addition, statins increase prostate-restricted replication component adenovirus (PRRA) replication, CAR, integrin, and death receptor 4 (DR4), leading to cholesterol depletion and increased TRAIL sensitivity [149]. Activation of PI3K/Akt signaling causes breast cancer resistance to MEK inhibition therapy by CH5126766 or trametinib. Treatment with simvastatin or fluvastatin overcomes this resistance by suppressing PI3K/Akt signaling and upregulating TRAIL [150]. In addition, sensitivity to sorafenib is enhanced by the inhibition of toll-like receptor 4 (TLR4)-mediated MAPK and NF-κB activation [121]. In addition, apoptotic resistance to MEK inhibitors is overcome by the inhibition of Akt activation and induction of TNF-related apoptosis-inducing ligand (TRAIL) [150].

### 5.9. Prednisolone

Although pitavastatin is considered to exhibit potential for use in the treatment of ovarian cancers, the necessity of a high dose of statin increases the risk of myopathy, the most common adverse effect associated with statins. Therefore, the reduction of statin dose is mandatory. Prednisolone was observed to be effective in synergizing the anti-cancer effect of pitavastatin. The use of pitavastatin combined with prednisolone decreases the expression of genes involved in the mevalonate pathway, such as mevalonate decarboxylase (MVD), farnesyl diphosphate synthase (FDPS), geranylgeranyltransferase I and II (GGTI, GGTII), and isopentenyl diphosphate isomerase (IDI1) [151].

**Table 2:** In vitro study of statins showing the synergistic action in combination with anti-cancer drugs to overcome anti-cancer therapy resistance.

Cancer Types/Cells	Statin	Concurrent Therapy	Statin Dose	Pathway	Ref
Colorectal cancer	Lovastatin	-	2 μM	Inhibits DNMT and demethylates the BMP2, TIMP3, and HIC1 promoters	[61]
Breast cancer		-	4,8,16 μM	Cell cycle arrest at G(0)/G (1) phase	[62]
MDA-MB-231 breast cancer		-	1–10 μM	Upregulates Raf1, amyloid β, MEK6, STAT1, myelin-oligodendrocyte glycoprotein, Vitamin D3 receptor, downregulates CREB, and γ glutamyl transferase	[63]
Glioblastoma		Gefitinib	10 μM	Decreases Akt	[126]
Human cholangiosarcoma		Gefitinib	5–10 μM	Increases cell cycle arrest, TNF-alpha, and decreases LKB1 activation	[127]
Human non-small cell lung carcinoma		Gefitinib	1–5 μM2	Increases PARP, caspase-3, decreases Bcl-2, RAS, p-RAF, p-ERK1/2, p-AKT, and p-EGFR	[129]
Chronic myeloid leukemia		Imatinib	5–20 μM	Decreases ABCB1 and ABCG2	[134]
Gall bladder cancer		Cisplatin	10–50 μM	Impairs DNA damage response	[137]
Prostate cancer		TRAIL	5 μM	Increases PRRA replication, CAR, and integrin	[149]
Glioblastoma		Temozolomide	0.625–20 μM	Impairs autophagy flux	[152]
Anaplastic thyroid cancer		Troglitazone	1–100 μM	Increases cell cycle arrest, p21, and p27	[153]
Multiple myeloma	Pravastatin	-	0.3, 0.6, and 0.9 μM	Increases cells in G0/G1 phase of the cell cycle and reduces the factors VEGF, and bFGF	[70]
Human hepatoma		-		Decreases p38 activity and expressions of p-p38, RhoC, and MMP-2, while elevates MKP-1 expression	[71]
Esophageal cancer	Simvastatin	-	0.625, 1.25, 2.5, 5, and 10 μM	Inhibits PTEN-PI3K/AKT pathway	[69]
Cholangiocarcinoma		-	1–100 μM, 25–50 μM	Reduces Rac1 activity, lowers expression of ABCA1 and ABCG1	[65,66]
Prostate cancer		Docetaxel	25 μM	Increases Bad, reduces Bcl-2, Bcl-xL and cleaved caspases 9/3, increases TNF, Fas-L, Traf1, and cleaved caspase 8	[64]
Prostate cancer		Doxorubicin	2.5–20 μM	Decreases ABGC4 protein	[98]
Malignant mesothelioma		Doxorubicin	10 μM	Increases NF-kB and NO production	[102]
Colon carcinoma		5-FU	5 mg/kg	Decreases tumor angiogenesis, Bcl-2 and increases Bax	[116]
Human salivary adenoid cystic carcinoma		MiR-21 inhibitor (miR-21i)	1–100 μM	Decreases N-Cadherin and increases E-Cadherin, decreases in Bcl-2 and survivin, while increase in p53, Bax, and caspase-9	[67]
Pancreatic ductal carcinoma	Simvastatin	Gemcitabine	5–40 μM	Increases Gfi-1, decreases CTGF	[144]
Chronic myeloid leukemia		Imatinib	10–50 μM	Increases cell cycle arrest, decreases STAT5 and STAT3	[135]
Metastatic melanoma		Dacarbazine	0.5–1 μM	Decreases RhoA/RhoC/LIM, increases p53, p21, p27, casp-3, and PARP	[154]
Breast cancer		Pentoxifylline	0.1–50 μM	Increases apoptosis, autophagy, and cell cycle arrest	[155]
Prostate cancer		Castration	0.1–20 μM	Increases cell cycle arrest, apoptosis, and decreases Akt	[156]
Blood cancer		Ventoclax	5–20 μM	Increases p53, PUMA	[157]
Non-small cell lung cancer		Gefitinib, Erlotinib	5 μM	Decreases Akt, b-catenin, survivin, cyclin D1	[130]
Gastric cancer xenograft		Capecitbine	10–50 μM	Decreases NF-kB	[117]
Melanoma cells		5,6-dimethylsanthenone-4-acetic acid	1.5–14 μM	Decreases HIF-alpha	[158]
Breast cancer		Anti-HER2	1–5 μM	Decreases YAP/TAZ signaling	[159]
Colon cancer	Simvastatin + phenothiazines	Doxorubicin	2.5 μM	Decreases ABCB1, COX-2 enzymes, Bcl-2 and increases Bax	[103]
Human myeloid leukemia	Simvastatin, Mevastatin, Lovastatin, Pravastatin	Doxorubicin, Paclitaxel, 5-FU	5–50 μM	Decreases NF-kB	[109]
Pancreatic cancer	Simvastatin + bisphosphonates	Gemcitabine	0.1–100 μM	Decreases cell viability	[141]
Colon cancer	Simvastatin + Oxicam derivatives	Doxorubicin	5 μM	Increases caspase-3, Bax, decreases Bcl-2 and COX-2	[107]
Prostate cancer	Simvastatin + Valproic acid	Docetaxel	1–100 μM	Decreases YAP	[160]
Acute myeloid leukemia (AML),	Fluvastatin	Tyrosine kinase inhibitor (lestaurtinib)	0.2–2 μM	Inhibits FLT3 glycosylation	[73]
C6 glioma cell line		-	1 to 10 μM	Decreases p-ERK1/2 expression, upregulates p-JNK1/2, and reduces MMP-9 and VEGF concentrations	[76]
Breast cancer		-	5–20 μM	Downregulates vimentin,	[79]
Breast cancer		-	10 μM	Increases p53 and induces autophagy	[81]
Human hepatoma cells (HepG2)		Trans-activator transcription peptide (TAT)	1–1000 μM	Accumulates cells in the pre-G phase	[82]
Melanoma cells		Sorafenib	1 μM	Increases PARP, and JNK	[119]
Hepatocellular carcinoma		Sorafenib	10 mg/kg	Inactivates MAPK and NF-kB	[121]
Melanoma cells		Vemurafenib	1–10 μM	Decreases Akt	[148]
Cervical cancer	Fluvastatin, Atorvastatin, and simvastatin	-	10–160 μM	Increases ROS and nitrite production	[75]
Lymphoma cells	Fluvastatin, atorvastatin, and simvastatin	-	0–5 μM	Enhances the DNA fragmentation and the activation of proapoptotic members such as caspase-3, PARP and Bax, increases reactive oxygen species (ROS), p38 MAPK activation but suppresses activation of anti-apoptotic molecule Bcl-2, decrease mitochondrial membrane potential and activation of Akt and Erk pathways	[78]
Human breast cancer	Fluvastatin and atorvastatin	Estradiol	-	Deregulates Bcl-2 rather than up-regulation of Fas-L or p53	[74]
Breast cancer	Fluvastatin and simvastatin	-	10 to 20 μM	Increases nitric oxide levels via iNOS expression, increases MnSOD, catalase and GSH which in turn, diminished H2O2 levels, down regulates transferrin receptor (TfR1), TfR1, MMP-2, 9	[79]
glioblastoma cell lines	Fluvastatin, cerivastatin, and pitavastatin	-	IC50 value: Ceri:0.0010 μM Pita:0.0023 μM Flu:0.109 μM	Increase autophagy	[80]
Breast cancer	Fluvastatin and simvastatin	CH51126766 or trametinib	0.3 μM	Decreases Akt and increases PARP	[150]
Non-small cell lung cancer	Fluvastatin and pitavastatin	Erlotinib	100 μM	Increases casp-3 and PARP	[133]
Cervical cancer	Fluvastatin, atorvastatin, and simvastatin	-	10–160 μM	Increases ROS and nitrite production	[75]
Lymphoma cells		-	0–5 μM	Enhances DNA fragmentation, caspase-3, PARP and Bax, but suppresses Bcl-2, increases reactive oxygen species (ROS) and activation of p38 MAPK, decreases mitochondrial membrane potential and activation of Akt and Erk pathways	[78]
NCI-H332M, DU-145, PC-3 and HOP-92 cell lines	Atorvastatin	-	0–30 μM	Inhibits protein prenylation	[83]
Human osteosarcoma		Doxorubicin and cisplatin	10 μM	Decreases MMP2	[100]
Hepatocellular carcinoma		Hypoxia	1–10 μM	Inactivates YAP	[120]
Human cholangiocarcinoma		Gemcitabine	5–100 μM	Decreases Yes-associated protein	[142]
Non-small cell lung cancer		Gefitinib	1–5 μM	Decreases Akt and ERK	[132]
Melanoma cancer		Tamoxifen	1–100 μM	Increases Bax and cytochrome C	[161]
Colon cancer		Celecoxib	15–45 μM	Increases cell cycle arrest and apoptosis	[162]
Prostate cancer	Rosuvastatin	-	5–50 μM	Decreases Vimentin and Zeb-1, and inhibits spheroid formation	[84]
Hepatic cancer		NA	The IC50 values ranged from 12 to 112 μg/mL	Enhances apoptosis and induces cell cycle arrest at G2/M phase	[87]
Murine mammary adenocarcinoma		Nilotinib	7.5 mg/kg	Increases caspase 3, decreases ER alpha, and tumor nitric oxide level	[163]
Hepatocellular carcinoma		Dasatinib	10, 25, 50 μM	Decreases p-FAK/p-Src, p-Ras/p-Raf, p-STAT3, p-Akt, HGF, VEGF, MMP-9, and Ki67	[164]
Adrenocortical carcinoma		Mitotane	100 μM	Decreases cell viability, ABCA1 and induces apoptosis	[165]
Ovarian cancer	Pitavastatin	-	1 μM	Increases caspase activity and apoptotic cell death	[89]
Oral squamous cell carcinoma		-	0.05–0.25 μM	Increases p-AMPK, FOXO3a, and PUMA while decreases p-Akt	[90]
Breast and melanoma model		Radiation	1.25, 2.5 or 5 μM	Increases senescence and delays DNA repair	[91]
Pancreatic ductal carcinoma		Gemcitabine	0.5 μM	Increases caspase-3, PARP, RIP1-RIP3-MLKL complex, decreases cyclineA2/CDK2, increases p21	[143]
Melanoma		Dacarbazine	1 μM	Increases apoptosis and autophagy cell death	[166]
Breast cancer	Cerivastatin	-	25 ng/mL	Down-regulates cyclin D1, PCNA, c-myc, and up-regulates p21, p19INK4d, integrin h8, (decrease in u-PA, MMP-9, u-PAR, PAI-1 and increase in anti-oncogenes Wnt-5a and H-cadherin	[92]
Human glioblastoma		-	10–100 μM	Down-regulates tyrosine phosphorylation of FAK	[93]
Breast cancer		NA	25 ng/mL	Induces cell cycle arrest at G1/S, inactivates Rho, NF-kB, and decreases MMP-9	[94]
Breast cancer		Doxorubicin and cisplatin	0.0195–0.624 μM	Increases p21	[101]
Colorectal cancer		5-FU	0.01–10 μM	Decreases nuclear factor kB binding activity	[115]
Malignant mesothelioma	Mevastatin	Doxorubicin	100 μM	Increases NF-kB and NO production	[102]

## 6. The Dark Side of Statin Therapy (Resistance and Intolerance to Statins)

Although statins have demonstrated anti-cancer effects under different conditions, they produce an unexpected effect in cancer treatment. Statin itself can induce resistance. The effect of statins on the regulation of multidrug resistance proteins was studied in vitro in hepatocytes, where statins (pitavastatin) help in the excretion of endogenous and exogenous lipophilic compounds from hepatocytes via multidrug resistance protein 2 (MDR2) expression [167]. Another in vitro study shows that fluvastatin causes resistance in cells comprised of hepatitis C virus replicon via an increase in HMG-CoA reductase and P-gp expression [168]. In prostate cancer xenograft model, combined treatment with a lipid-lowering drug, ezetimibe, and simvastatin increases tumor growth along with the induction of low-density lipoprotein (LDL) receptor; however, the serum cholesterol level decreases, suggesting that the induction of LDL receptor can be a possible mechanism of resistance development under these treatments [169]. The insensitivity of breast cancer cells to atorvastatin treatment was determined to be due to increased unsaturated fatty acid metabolism and cholesterol biosynthesis through the induction of stearoyl-CoA desaturase (SCD) and 3-hydroxy03-methylglutaryl-CoA reductase (HMGCR), respectively [170]. Breast cancer cells show different vulnerabilities to statins (atorvastatin, simvastatin, and rosuvastatin). For example, breast cancer cells, such as MDA-MB-231 and MDA-MB-468, show sensitivity, while T47D and MCF-7 cells show resistance. The development of statin resistance in T47D and MCF-7 cells is due to sterol regulatory element-binding protein (SREBP-2)-mediated induction of HMGCR mRNA and protein expression [171]. Along with the induction of cytotoxicity in breast cancer cells, lovastatin causes autophagy flux, as well as induction of the multidrug resistance proteins MDR1 and TGF-β1, indicating the possibility of resistance development. However, the inhibition of autophagy flux by treatment with chloroquine decreased the expression of MDR1 and TGF-β1, demonstrating that lovastatin can cause breast cancer cell resistance by inducing the autophagy flux [172]. The clinical study shows that statin resistance and intolerance are associated with polymorphisms in genes such as HMG-CoA reductase, TNF-α, BCRP/ABCG2, P-gp/ABCB1, MRP1/ABCC1, MRP2/ABCC2, CETP, ApoE, PCSK9, RHOA, CYP7A1, LDLR, LPA, OATP, NPC1L1, and FXR [173].

## 7. Statin-Mediated Resistance

One of the mechanisms through which statins induce resistance development is the expression of MDR2 proteins. For example, the activation of the liver X-receptor (LXR) α/β, SREBP-1, and HMGCR expression have been involved leading to the induction of MDR2 [167,171]. The induction of the LDL receptor is another factor to increase tumor growth, along with the development of resistance [169]. The induction of the stearoyl-CoA desaturase (SCD) and HMGCR under statin treatment leads to resistance by an increase in unsaturated fatty acid metabolism and cholesterol biosynthesis [170]. Furthermore, according to clinical data polymorphisms of the genes such as HMG-CoA reductase, TNF-α, BCRP/ABCG2, P-gp/ABCB1, MRP1/ABCC1, MRP2/ABCC2, CETP, ApoE, PCSK9, RHOA, CYP7A1, LDLR, LPA, OATP, NPC1L1, and FXR have been linked to statin resistance an intolerance [173].

## 8. A Clinical Trial of Statin in Cancer

The epidemiologic study has proved that the use of hydrophobic statin (simvastatin, lovastatin, Fluvastatin) but not hydrophilic statins (pravastatin and atorvastatin) have been associated with the reduced breast cancer risk [174]. In a continuing study, the first clinical trial of simvastatin in refractory multiple myeloma was done in 2007. Simvastatin was administered to refractory multiple myeloma patients with the concomitant administration of two cycles of bortezomib or bendamustine. The use of simvastatin was well tolerated without grade 3/4 toxicity, and patients administered with simvastatin showed a reduction in bortezomib or bendamustine resistance through inhibition of HMG-CoA reductase [46], suggesting that the simvastatin helps to improve the efficacy of bortezomib or bendamustine in these conditions. A phase II clinical trial was performed in metastatic colorectal cancer patients with KRAS mutations for evaluating the safety and efficacy of the combination of simvastatin with cetuximab in patients who were previously exposed to fluoropyrimidine, oxaliplatin, and irinotecan. The clinical data showed that only 4 out of 18 (22.2%) patients were free from progression at the primary end point at 20.3 to 47 weeks [48].

The clinical study of other statins, i.e., atorvastatin and fluvastatin, was conducted in 9135 subjects with chronic hepatitis C virus (HCV) from 2001 to 2014, and the result shows that the use of statins reduced the risk of fibrosis progression and caused a 49% reduction in the incidence of hepatocellular carcinoma. The reduction of HCC by statin is believed to be due to the inhibition of thioredoxin, a hepatic enzyme, increased in pre-malignant hepatic nodule to mediate cell survival [175].

A pilot window-of-opportunity study of fluvastatin shows that in 33 men with prostate cancer, the use of fluvastatin before radical prostatectomy (RP) increased prostate cancer cell death with an increase in cleaved caspase-3 without alteration in intratumoral ki67, a marker of cancer [50], suggesting that the use of fluvastatin prior to RP improves the effect on tumor cells apoptosis. The phase I clinical trial was performed to determine the safety and dose of rosuvastatin in patients with advanced solid malignancies in which a dose of rosuvastatin (1–8 mg/kg/day) in combination with erlotinib was used. Although the combination has shown the observed disease stabilization rate of 25%, the high level of muscle toxicities including fatigue, muscle weakness, and myalgia have been detected limiting the use of rosuvastatin in combination with erlotinib [52]. However, a randomized, single-blind, placebo-controlled trial has indicated that rosuvastatin can be used to inhibit chemotherapy-induced cardiotoxicity in woman with breast cancer [53].

In a phase II clinical trial, a combination of pravastatin with sorafenib was safe and well-tolerated with prolonged time to progression (TTP) in advanced hepatocellular carcinoma [123]. A pooled analysis of metastatic renal cell carcinoma (mRCC) treated on phase II and phase III clinical trials by sunitinib, sorafenib, axitinib, temsirolimus, temsirolimus + interferon (IFN)-α, bevacizumab + temsirolimus, bevacizumab + IFN-α, or IFN-α, along with or without statin were evaluated with overall survival. The data showed that the use of statins along with other anti-cancer adjuvants increased the overall survival to 25.6 months compared to treatment without statins, which resulted in survival of 18.9 months [176]. Further, the retrospective study showed that the use of statin is associated with improved overall survival in gemcitabine–erlotinib combination chemotherapy in patients with advanced pancreatic cancer [47]. A nationwide, population-based case-control study conducted in Taiwan in lung cancer patients receiving EGFR-TKIs therapy has shown that the use of statin reduces the risk of death with significant increase in median progression-free survival (8.3 months vs 6.1 months) and median overall survival (35.5 months vs. 23.9 months) [177]. However, certain clinical studies have demonstrated the inefficient use of statins in clinical trials. For example, the use of pravastatin together with cyclosporine A, mitoxantrone, and etoposide induced excessive toxicity and failed to achieve acceptable efficacy in phase I and II clinical trials [178]. Moreover, the use of statins in combination with other chemotherapeutic drugs improves neither progression-free survival nor overall survival [179]. Collectively, these results suggest that additional research is needed to confirm the safe and effective use of statins as an adjuvant in cancer therapy.

## 9. Conclusions

Here, we discussed the use of statins in different anti-cancer therapies. Statin alone has been used as an anti-proliferative and apoptotic action for in vitro studies in combination with other anti-cancer drugs for its synergistic action, or alone or in combination with other anti-cancer drugs for overcoming drug resistance induced by certain cancer therapies. Furthermore, we discussed the possibilities of inhibiting signaling pathways or signaling molecules by statins under different cancer therapies in different cancer cells. Statins show potential effects—either alone or in combination—on the basis of the type of cancer cells and gene mutation status. However, the use of statins as adjuvants, either alone or in combination, appears to benefit cancer patients in the context of drug repurposing to reduce the cost.

## Data Availability

No new data were created or analyzed in this study. Data sharing is not applicable to this article.
